# Support Vector Machine Implementations for Classification & Clustering

**DOI:** 10.1186/1471-2105-7-S2-S4

**Published:** 2006-09-26

**Authors:** Stephen Winters-Hilt, Anil Yelundur, Charlie McChesney, Matthew Landry

**Affiliations:** 1 Department of Computer Science, University of New Orleans, New Orleans, LA, 70148, USA; 2 The Research Institute for Children, 200 Henry Clay Ave., New Orleans, LA 70118, USA

## Abstract

**Background:**

We describe Support Vector Machine (SVM) applications to classification and clustering of channel current data. SVMs are variational-calculus based methods that are constrained to have structural risk minimization (SRM), i.e., they provide noise tolerant solutions for pattern recognition. The SVM approach encapsulates a significant amount of model-fitting information in the choice of its kernel. In work thus far, novel, information-theoretic, kernels have been successfully employed for notably better performance over standard kernels. Currently there are two approaches for implementing multiclass SVMs. One is called external multi-class that arranges several binary classifiers as a decision tree such that they perform a single-class decision making function, with each leaf corresponding to a unique class. The second approach, namely internal-multiclass, involves solving a single optimization problem corresponding to the entire data set (with multiple hyperplanes).

**Results:**

Each SVM approach encapsulates a significant amount of model-fitting information in its choice of kernel. In work thus far, novel, information-theoretic, kernels were successfully employed for notably better performance over standard kernels. Two SVM approaches to multiclass discrimination are described: (1) internal multiclass (with a single optimization), and (2) external multiclass (using an optimized decision tree). We describe benefits of the internal-SVM approach, along with further refinements to the internal-multiclass SVM algorithms that offer significant improvement in training time without sacrificing accuracy. In situations where the data isn't clearly separable, making for poor discrimination, signal clustering is used to provide robust and useful information – to this end, novel, SVM-based clustering methods are also described. As with the classification, there are Internal and External SVM Clustering algorithms, both of which are briefly described.

## Background

### Support Vector Machine

**Figure 1:**
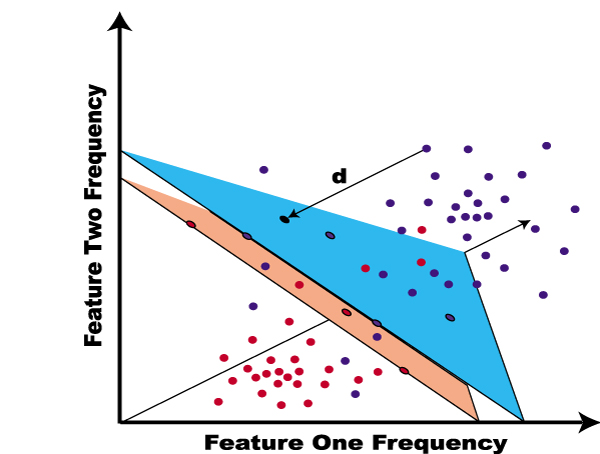
A sketch of the hyperplane separability heuristic for SVM binary classification. An SVM is trained to find an optimal hyperplane that separates positive and negative instances, while also constrained by structural risk minimization (SRM) criteria, which here manifests as the hyperplane having a thickness, or "margin," that is made as large as possible in seeking a separating hyperplane. A benefit of using SRM is much less complication due to overfitting (a common problem with Neural Network discrimination approaches). Given its geometric expression, it is not surprising that a key construct in the SVM formulation (via the choice of kernel) is the notion of "nearness" between instances (or nearness to the hyperplane, where it gives a measure of confidence in the classification, i.e., instances further from the decision hyperplane are called with greater confidence). Most notions of nearness explored in this context have stayed with the geometric paradigm and are known as "distance kernels," one example being the familiar Gaussian kernel which is based on the Euclidean distance: K_Gaussian_(x,y) = exp(-D_Eucl._(x,y)^2^/2σ^2^), where D_Eucl._(x,y) = [∑_k_(x_k_-y_k_)2]^1/2 ^is the usual Euclidean distance. Those kernels are used in the signal pattern recognition analysis in Figure 8 along with a new class of kernels, "divergence kernels," based on a notion of nearness appropriate when comparing probability distributions (or probability feature vectors). The main example of this is the Entropic Divergence Kernel: K_Entropic _= exp(-D_Entropic._(x,y)^2^/2σ^2^), where D_Entropic._(x,y) = D(x||y) + D(y||x) and D(..||..) is the Kullback-Leibler Divergence (or relative entropy) between x and y.

SVMs are fast, easily trained, discriminators [1,2], for which strong discrimination is possible without the over-fitting complications common to neural net discriminators [1]. SVMs strongly draw upon variational methods in their construction and are designed to yield the best estimate of the optimal separating hyperplane (for classifier, see Fig. 1) with confidence parameter information included (via hyperplane with margin optimization used in structural risk minimization). The SVM approach also encapsulates a significant amount of model fitting and discriminatory information in the choice of kernel in the SVM, and a number of novel kernels have been developed. In [3], novel, information-theoretic, kernels were introduced for notably better performance over standard kernels (with discrete probability distributions as part of feature vector data). The classification approach adopted in [3] is designed to scale well to multi-species classification (or a few species in a very noisy environment). The scaling is possible due to use of a decision tree architecture and an SVM approach that permits rejection on weak data. SVMs are usually implemented as binary classifiers, are in many ways superior to neural nets, and may be grouped in a decision tree to arrive at a multi-class discriminator. SVMs are much less susceptible to over-training than neural nets, allowing for a much more hands-off training process that is easily deployable and scalable. A multiclass implementation for an SVM is also possible – where multiple hyperplanes are optimized simultaneously. A (single-optimization, multi-hyperplane) multiclass SVM has a much more complicated implementation, but the reward is a classifier that is much easier to tune and train, especially when considering data rejection. The (single) multiclass SVM, doesn't have as non-scalable a throughput problem (with tree depth), and even appears to offer a natural drop zone via its margin definition, so is being considered in further refinements of the method.

SVMs use variational methods in their construction and encapsulate a significant amount of discriminatory information in their choice of kernel. In reference [3] information-theoretic kernels provided notably better performance than standard kernels. Feature extraction was designed to arrive at probability vectors (i.e., discrete probability distributions) on a predefined, and complete, space of possibilities. (The different blockade levels, and their frequencies, the emission probabilities, and the transition probabilities, for example.) This turns out to be a very general formulation, wherein feature extraction makes use of signal decomposition into a complete set of separable states that can be interpreted or represented as a probability vector. A probability vector formulation also provides a straightforward hand-off to the SVM classifiers since all feature vectors have the same length with such an approach. What this means for the SVM, however, is that geometric notions of distance are no longer the best measure for comparing feature vectors. For probability vectors (i.e., discrete distributions), the best measures of similarity are the various information-theoretic divergences: Kullback-Leibler, Renyi, etc. By symmetrizing over the arguments of those divergences a rich source of kernels is obtained that works well with the types of probabilistic data obtained.

The SVM discriminators are trained by solving their KKT relations using the Sequential Minimal Optimization (SMO) procedure [4]. A chunking [5,6] variant of SMO also is employed to manage the large training task at each SVM node. The multi-class SVM training generally involves thousands of blockade signatures for each signal class. The data cleaning needed on the training data is accomplished by an extra SVM training round.

### Binary Support Vector Machines

Binary Support Vector Machines (SVMs) are based on a decision-hyperplane heuristic that incorporates structural risk management by attempting to impose a training-instance void, or "margin," around the decision hyperplane [1].

Feature vectors are denoted by x_ik_, where index i labels the M feature vectors (1 ≤ i ≤ M) and index k labels the N feature vector components (1 ≤ i ≤ N). For the binary SVM, labeling of training data is done using label variable y_i _= ± 1 (with sign according to whether the training instance was from the positive or negative class). For hyperplane separability, elements of the training set must satisfy the following conditions: w_β_x_iβ_-b ≥ +1 for i such that y_i _= +1, and w_β_x_iβ_-b ≤ -1 for y_i _= -1, for some values of the coefficients w_1_, ..., w_N_, and b (using the convention of implied sum on repeated Greek indices). This can be written more concisely as: y_i_(w_β_x_iβ_-b) - 1 ≥ 0. Data points that satisfy the equality in the above are known as "support vectors" (or "active constraints").

Once training is complete, discrimination is based solely on position relative to the discriminating hyperplane: w_β_x_iβ _- b = 0. The boundary hyperplanes on the two classes of data are separated by a distance 2/w, known as the "margin," where w^2 ^= w_β_w_β_. By increasing the margin between the separated data as much as possible the optimal separating hyperplane is obtained. In the usual SVM formulation, the goal to maximize w^-1 ^is restated as the goal to minimize w^2^. The Lagrangian variational formulation then selects an optimum defined at a saddle point of L(w,b;α) = (w_β_w_β_)/2 - α_γ_y_γ_(w_β_x_γβ_-b) - α_0_, where α_0 _= Σ_γ_α_γ_, α_γ _≥ 0 (1 ≤ γ ≤ M). The saddle point is obtained by minimizing with respect to {w_1_, ...,w_N_,b} and maximizing with respect to {α_1_, ..., α_M_}. If y_i_(w_β_x_iβ_-b) - 1 ≥ 0, then maximization on α_i _is achieved for α_i _= 0. If y_i_(w_β_x_iβ_-b) - 1 = 0, then there is no constraint on α_i_. If y_i_(w_β_x_iβ_-b) - 1 < 0, there is a constraint violation, and α_i _→ ∞. If absolute separability is possible the last case will eventually be eliminated for all α_i_, otherwise it's natural to limit the size of α_i _by some constant upper bound, i.e., max(α_i_) = C, for all i. This is equivalent to another set of inequality constraints with α_i _≤ C. Introducing sets of Lagrange multipliers, ξ_γ _and μ_γ_(1 ≤ γ ≤ M), to achieve this, the Lagrangian becomes:

L(w,b;α,ξ,μ) = (w_β_w_β_)/2 - α_γ_[y_γ_(w_β_x_γβ_-b)+ξ_γ_] + α_0 _+ ξ_0_C - μ_γ_ξ_γ_, where ξ_0 _= Σ_γ_ξ_γ_, α_0 _= Σ_γ_α_γ_, and α_γ _≥ 0 and ξ_ξ _≥ 0 (1 ≤ γ ≤ M).

At the variational minimum on the {w_1_, ...,w_N_,b} variables, w_β _= α_γ_y_γ_x_γβ_, and the Lagrangian simplifies to: L(α) = α_0 _- (α_δ_y_δ_x_δβ _α_γ_y_γ_x_γβ_/2, with 0 ≤ α_γ _≤ C (1 ≤ γ ≤ M) and α_γ_y_γ _= 0, where only the variations that maximize in terms of the α_γ _remain (known as the Wolfe Transformation). In this form the computational task can be greatly simplified. By introducing an expression for the discriminating hyperplane: f_i _= w_β_x_iβ _- b = α_γ_y_γ_x_γβ_x_iβ _- b, the variational solution for L(α) reduces to the following set of relations (known as the Karush-Kuhn-Tucker, or KKT, relations): (i) α_i _= 0 ⇔ y_i_f_i _≥ 1, (ii) 0 < α_i _< C ⇔ y_i_f_i _= 1, and (iii) α_i _= C ⇔ y_i_f_i _≤ 1. When the KKT relations are satisfied for all of the α_γ _(with α_γ_y_γ _= 0 maintained) the solution is achieved. (The constraint α_γ_y_γ _= 0 is satisfied for the initial choice of multipliers by setting the α's associated with the positive training instances to 1/N^(+) ^and the α's associated with the negatives to 1/N^(-)^, where N^(+) ^is the number of positives and N^(-) ^is the number of negatives.) Once the Wolfe transformation is performed it is apparent that the training data (support vectors in particular, KKT class (ii) above) enter into the Lagrangian solely via the inner product x_iβ_x_jβ_. Likewise, the discriminator f_i_, and KKT relations, are also dependent on the data solely via the x_iβ_x_jβ _inner product.

Generalization of the SVM formulation to data-dependent inner products other than x_iβ_x_jβ _are possible and are usually formulated in terms of the family of symmetric positive definite functions (reproducing kernels) satisfying Mercer's conditions [1].

### Binary SVM Discriminator Implementation

The SVM discriminators are trained by solving their KKT relations using the Sequential Minimal Optimization (SMO) procedure of [4]. The method described here follows the description of [4] and begins by selecting a pair of Lagrange multipliers, {α_1_,α_2_}, where at least one of the multipliers has a violation of its associated KKT relations (for simplicity it is assumed in what follows that the multipliers selected are those associated with the first and second feature vectors: {x_1_,x_2_}). The SMO procedure then "freezes" variations in all but the two selected Lagrange multipliers, permitting much of the computation to be circumvented by use of analytical reductions:

L(α_1_,α_2_;α_β'≥3_) = α_1 _+ α_2 _- (α_1_^2^K_11 _+ α_2_^2^K_22 _+ 2α_1_α_2_y_1_y_2_K_12_)/2 - α_1_y_1_v_1 _- α_2_y_2_v_2 _+ α_β'_U_β' _- (α_β'_α_γ'_y_β'_K_β'γ'_)/2,

with β',γ' ≥ 3, and where K_ij _≡ K(x_i_, x_j_), and v_i _≡ α_β'_y_β'_K_iβ' _with β' ≥ 3. Due to the constraint α_β_y_β _= 0, we have the relation: α_1 _+ sα_2 _= -γ, where γ ≡ y_1_α_β'_y_β' _with β' ≥ 3 and s ≡ y_1_y_2_. Substituting the constraint to eliminate references to α_1_, and performing the variation on α_2_: ∂L(α_2_;α_β'≥3_)/∂α_2 _= (1 - s) + ηα_2 _+ sγ(K_11 _- K_22_) + sy_1_v_1 _- y_2_v_2_, where η ≡ (2K_12 _- K_11 _+ K_22_). Since v_i _can be rewritten as v_i _= w_β_x_iβ _- α_1_y_1_K_i1 _- α_2_y_2_K_i2_, the variational maximum ∂L(α_2_;α_β'≥3_)/∂α_2 _= 0 leads to the following update rule:

α_2_^new ^= α_2_^old ^- y_2_((w_β_x_1β_-y_1_) - (w_β_x_2β_-y_2_))/η.

Once α_2_^new ^is obtained, the constraint α_2_^new ^≤ C must be re-verified in conjunction with the α_β_y_β _= 0 constraint. If the L(α_2_;α_β'≥3_) maximization leads to a α_2_^new ^that grows too large, the new α_2 _must be "clipped" to the maximum value satisfying the constraints. For example, if y_1 _≠ y_2_, then increases in α_2 _are matched by increases in α_1_. So, depending on whether α_2 _or α_1 _is nearer its maximum of C, we have max(α_2_) = argmin{α_2_+(C-α_2_); α_2_+(C-α_1_)}. Similar arguments provide the following boundary conditions: (i) if s = -1, max(α_2_) = argmin{α_2_; C+α_2_-α_1_}, and min(α_2_) = argmax{0; α_2_-α_1_}, and (ii) if s = +1, max(α_2_) = argmin{C; α_2_+α_1_}, and min(α_2_) = argmax{0; α_2_+α_1_-C}. In terms of the new α_2_^new, clipped^, clipped as indicated above if necessary, the new α_1 _becomes:

α_1_^new ^= α_1_^old ^+ s(α_2_^old^-α_2_^new, clipped^),

where s ≡ y_1_y_2 _as before. After the new α_1 _and α_2 _values are obtained there still remains the task of obtaining the new b value. If the new α_1 _is not "clipped" then the update must satisfy the non-boundary KKT relation: y_1_f(x_1_) = 1, i.e., f^new^(x_1_) - y_1 _= 0. By relating f^new ^to f^old ^the following update on b is obtained:

b^new1 ^= b - (f^new^(x_1_) - y_1_) - y_1_(α_1_^new - α^_1_^old^)K_11 _- y_2_(α_2_^new, clipped ^- α_2_^old^)K_12_.

If α_1 _is clipped but α_2 _is not, the above argument holds for the α_2 _multiplier and the new b is:

b^new2 ^= b - (f^new^(x_2_) - y_2_) - y_2_(α_2_^new - α^_2_^old^)K_22 _- y_1_(α_1_^new, clipped ^- α_1_^old^)K_12_.

If both α_1 _and α_2 _values are clipped then any of the b values between b^new1 ^and b^new2 ^is acceptable, and following the SMO convention, the new b is chosen to be:

b^new ^= (b^new1 ^+ b^new2^)/2.

### Multiclass SVM Methods

The SVM binary discriminator offers high performance and is very robust in the presence of noise. This allows a variety of reductionist multiclass approaches, where each reduction is a binary classification (for classifying cards by suit, maybe classify as red or black first, then as heart or diamond for red and spade or club for black, for example). The SVM Decision Tree is one such approach, and a collection of them (a SVM Decision Forest) can be used to avoid problems with throughput biasing. Alternatively, the variational formalism can be modified to perform a multi-hyperplane optimization situation for a direct multiclass solution [7-9], and that is what is described next.

### SVM-Internal Multiclass

In the formulation in [7], there are 'k' classes and hence 'k' linear decision functions – a description of their approach is given here. For a given input 'x', the output vector corresponds to the output from each of these decision functions. The class of the largest element of the output vector gives the class of 'x'.

Each decision function is given by: f_m_(x) = w_m_.x + b_m _for all m = (1,2, ..., k). If y_i _is the class of the input x_i_, then for each input data point, the misclassification error is defined as follows: max_m_{f_m_(x_i_) + 1 - δ_i_^m^} - f_yi_(x_i_), where δ_i_^m ^is 1 if m = y_i _and 0 if m ≠ y_i_. We add the slack variable ζ_i _where ζ_i _≥ 0 for all i that is proportional to the misclassification error: max_m_{f_m_(x_i_) + 1 - δ_i_^m^} - f_yi_(x_i_) = ζ_i_, hence f_yi_(x_i_) - f_m_(x_i_) + δ_i_^m ^≥ 1 - ζ_i _for all i, m. To minimize this classification error and maximize the distance between the hyper-planes (Structural Risk Minimization) we have the following formulation:

Minimize: ∑_i_ζ_i _+ β(1/2)∑_m_w_m_^T^w_m _+ (1/2)∑_m_b_m_^2^,

where β > 0 is defined as a regularization constant.

Constraint: w_yi_.x_i _+ b_yi _- w_m_.x_i _- b_m _- 1 + ζ_i _+ δ_i_^m ^≥ 0 for all i,m

Note: the term **(1/2)∑**_**m**_**b**_**m**_^**2 **^is added for de-coupling, 1/β = C, and m = y_i _in the above constraint is consistent with ζ_i _≥ 0. The Lagrangian is:

L(w,b,ζ) = ∑_i_ζ_i _+ β(1/2)∑_m_w_m_^T^w_m _+ (1/2)∑_m_b_m_^2 ^- ∑_i_∑_m_α_i_^m^(w_yi_x_i _+ b_yi _- w_m_.x_i _- b_m _- 1 + ζ_i _+ δ_i_^m^)

Where all α_i_^m^s are positive Lagrange multipliers. Now taking partial derivatives of the Lagrangian and equating them to zero (Saddle Point solution): ∂L/∂ζ_i _= 1 - ∑_m_α_i_^m ^= 0. This implies that ∑_m_α_i_^m ^= 1 for all i. ∂L/∂b_m _= b_m _+ ∑_i_α_i_^m ^- ∑_i_δ_i_^m ^= 0 for all m. Hence b_m _= ∑_i_(δ_i_^m ^- α_i_^m^). Similarly: ∂L/∂w_m _= βw_m _+ ∑_i_α_i_^m^x_i _- ∑_i_δ_i_^m^x_i _= 0 for all m. Hence w_m _= (1/β)[∑_i_(δ_i_^m ^- α_i_^m^)x_i_] Substituting the above equations into the Lagrangian and after simplification reduces into the dual formalism:

Maximize: -1/2∑_i,j_∑_m_(δ_i_^m ^- α_i_^m^)(δ_j_^m ^- α_j_^m^)(K_ij _+ β) - β∑_i,m_δ_i_^m^α_i_^m^

Constraint: 0 ≤ α_i_^m^, ∑_m_α_i_^m ^= 1, i = 1...1; m = 1...k

Where K_ij _= x_i_.x_j _is the Kernel generalization. In vector notation:

Maximize: -1/2∑_i,j_(Δ_yi _- A_i_)(Δ_yj _- A_j_)(K_ij _+ β) - β∑_i_Δ_yi_A_i_

Constraint: 0 ≤ A_i_, A_i_. 1 = 1, i = 1 ...1

Let τ_i _= Δ_yi _- A_i_. Hence after ignoring the constant: -1/2∑_i,j_τ_i_.τ_j_(K_ij _+ β) + β∑_i_Δ;_yi_τ_i_, subject to: τ_i _≤ Δ_yi_, τ_i_.1 = 0, i = 1 ...l. The dual is solved (determine the optimum values of all the τs) using the decomposition method.

Minimize: 1/2∑_i,j_τ_i_^m^.τ_j_^m^(K_ij _+ β) - β∑_i,m_δ_i_^m^τ_i_^m^

Constraint: τ_i _≤ Δ_yi_, τ_i_.1 = 0, i = 1 ...l

The Lagrangian of the dual is:

L = 1/2∑_i,j,m_τ_i_^m^.τ_j_^m^(K_ij _+ β) - φ∑_i,m_δ_i_^m^τ_i_^m ^- ∑_i,m_u_i_^m^(δ_i_^m ^- τ_i_^m^) - ∑_i_v_i_∑_m_τ_i_^m^

Subject to u_i_^m ^≥ 0

We take the gradient of the Lagrangian with respect to τ_i_^m^:

▼_τ_^m^[L] = ∑_i_τ_j_^m^(K_ij _+ β) - βδ_i_^m ^+ u_i_^m ^- v_i _= 0

Introducing f(τ) = ∑_i_τ_j_^m^(K_ij _+ β) - βδ_i_^m ^+ u_i_^m ^- v_i _= 0 and f_i_^m ^= ∑_i_τ_j_^m^(K_ij _+ β) - βδ_i_^m^, then f(τ) = f_i_^m ^+ u_i_^m ^- v_i _= 0. By KKT conditions we get two more equations:

u_i_^m^(δ_i_^m ^- τ_i_^m^) = 0 and u_i_^m ^≥ 0

Case I: if δ_i_^m ^= τ_i_^m^, then u_i_^m ^≥ 0, hence f_i_^m ^≤ v_i_. Case II: if τ_i_^m ^< δ_i_^m^, then u_i_^m ^= 0, hence f_i_^m ^= v_i_. Note: There is atleast one 'm' for all i such that τ_i_^m ^< δ_i_^m ^is satisfied.

Therefore combining Case I & II, we get:

max_m_{f_i_^m^} ≤ v_i _≤ min_m: τi_^m ^< δ_i_^m^{f_i_^m^}

Or max_m_{f_i_^m^} ≤ min_m: τi_^m ^< δ_i_^m^{f_i_^m^}

Or max_m_{f_i_^m^} - min_m: τi_^m ^< δ_i_^m^{f_i_^m^} ≤ ε

Note: τ_i_^m ^< δ_i_^m ^implies that α_i_^m ^> 0. Since ∑_m_α_i_^m ^= 1, for any i each α_i_^m ^is treated as the probability that the data point belongs to class m. Hence we define KKT violators as:

max_m_{f_i_^m^} - min_m: τi_^m ^< δ_i_^m^{f_i_^m^} > ε for all i.

### Decomposition Method to Solve the Dual

Using the method in [7] to solve the Dual, maximize

Q(τ) = -1/2∑_i,j_τ_i_.τ_j_(K_ij _+ β) + β∑_i_Δ_yi_τ_i_

Subject to: τ_i _≤ Δ_yi_, τ_i_.1 = 0, i = 1 ...l

Expanding in terms of a single 'τ' vector:

Q_p_(τ_p_) = -1/2A_p_(τ_p_. τ_p_) - B_p_.τ_p _+ C_p_

Where:

A_p _= K_pp _+ β

B_p _= -βΔ_yp _+ ∑_i≠p_τ_i_(K_ip _+ β)

C_p _= -1/2∑_i,j≠p_τ_i_.τ_j_(K_ij _+ β) + β∑_i≠p_τ_i_Δ_yi_

Therefore ignoring the constant term 'C_p_', we have to minimize:

Q_p_(τ_p_) = 1/2A_p_(τ_p_. τ_p_) + B_p_.τ_p_

Subject to: τ_p _≤ Δ_yp _and τ_p_.1 = 0

The above equation can also be written as:

Q_p_(τ_p_) = 1/2A_p_(τ_p _+ B_p_/A_p_).(τ_p _+ B_p_/A_p_) - B_p_.B_p_/2A_p_

Substitute v = (τ_p _+ B_p_/A_p_) & D = (Δ_yp _+ B_p_/A_p_) in the above equation. Hence, after ignoring the constant term B_p_.B_p_/2A_p _and the multiplicative factor 'A_p_' we have to minimize:

Q(v) = 1/2v.v = 1/2||v||^2^

Subject to: v ≤ D and v.1 = D.1 - 1

The Lagrangian is given by:

L(v) = 1/2||v||^2 ^- ∑_m_ρ_m_(D_m _- v_m_) - σ[∑_m_(v_m _- D_m_) + 1]

Subject to: ρ_m _≤ 0

Hence ∂L/∂v_m _= v_m _+ ρ_m _- σ = 0. By KKT conditions we have: ρ_m_(D_m _- v_m_) = 0 & ρ_m _≥ 0, also v_m _≤ D_m_. Hence by combining the above in-equalities, we have: v_m _= Min{D_m_, σ}, or ∑_m_v_m _= ∑_m_Min{D_m_, σ} = ∑_m_D_m _- 1. The above equation uniquely defines the 'σ' that satisfies the above equation AND that 'σ' is the optimal solution of the quadratic optimization problem. (Refer to [7] for a formal proof).

*Solve for 'σ': *We have Min{D_m_, σ} + Max{D_m_, σ} = D_m _+ σ, hence ∑_m_[D_m _+ σ - Max{D_m_, σ}] = ∑_m_D_m _- 1, or σ = 1/K[∑_m_Max{D_m_, σ} - 1], hence we find σ (iteratively) that satisfies the equation: |(σ_l _- σ_l+1_)/σ_l_| ≤ tolerance. The initial value for 'σ' is set to σ_1 _= 1/K[∑_m_D_m _- 1].

*Update rule for 'τ': *Once we have 'σ', τ_new_^m ^= v_m _- B_p_^m^/(K_pp _+ β), or:

τ_new_^m ^= v_m _- f_p_^m^/(K_pp _+ β) + τ_old_^m^

### SVM-Internal Clustering

Let {x_i_} be a data set of 'N' points in R^d^. Using a non-linear transformation φ, we transform 'x' to some high-dimensional space called Kernel space and look for the smallest enclosing sphere of radius 'R'. Hence we have: ||φ(x_j_) - a ||^2 ^≤ R^2 ^for all j = 1,...,N; where 'a' is the center of the sphere. Soft constraints are incorporated by adding slack variables 'ζ_j_':

||φ(x_j_) - a ||^2 ^≤ R^2 ^+ ζ_j _for all j = 1,...,N

Subject to: ζ_j _≥ 0

We introduce the Lagrangian as:

L = R^2 ^- ∑_j_β_j_(R^2 ^+ ζ_j _- ||φ(x_j_) - a ||^2^) - ∑_j_ζ_j_μ_j _+ C∑_j_ζ_j_

Subject to: β_j _≥ 0, μ_j _≥ 0,

where C is the cost for outliers and hence C∑_j_ζ_j _is a penalty term. Setting to zero the derivative of 'L' w.r.t. R, a and ζ we have: ∑_j_β_j _= 1; a = ∑_j_β_j_φ(x_j_); and β_j _= C - μ_j_.

Substituting the above equations into the Lagrangian, we have the dual formalism as:

W = 1 - ∑_i,j_β_i_β_j_K_ij _where 0 ≤ β_i _≤ C; K_ij _= exp(-||x_i _- x_j_||^2^/2σ^2^)

Subject to: ∑_i_β_i _= 1

By KKT conditions we have: ζ_j_μ_j _= 0 and β_j_(R^2 ^+ ζ_j _- ||φ(x_j_) - a ||^2^) = 0.

In the kernel space of a data point 'x_j_' if ζ_j _> 0, then β_j _= C and hence it lies outside of the sphere i.e. R^2 ^< ||φ(x_j_) - a ||^2^. This point becomes a bounded support vector or BSV. Similarly if ζ_j _= 0, and 0 < β_j _< C, then it lies on the surface of the sphere i.e. R^2 ^= ||φ(x_j_) - a ||^2^. This point becomes a support vector or SV. If ζ_j _= 0, and β_j _= 0, then R^2 ^> ||φ(x_j_) - a ||^2 ^and hence this point is enclosed with-in the sphere.

### Nanopore Detector based Channel Current Cheminformatics

**Figure 2:**
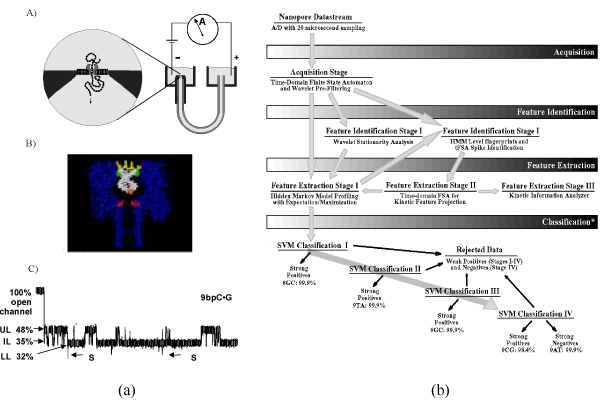
**a. **(A) shows a nanopore device based on the α-hemolysin channel. It has been used for analysis of single DNA molecules, such as ssDNA, shown, and dsDNA, a nine base-pair DNA hairpin is shown in (B) superimposed on the channel geometry. The channel current blockade trace for the nine base-pair DNA hairpin blockade from (B) is shown in (C). **b **shows the signal processing architecture that was used to classify DNA hairpins with this approach: Signal acquisition was performed using a time-domain, thresholding, Finite State Automaton, followed by adaptive pre-filtering using a wavelet-domain Finite State Automaton. Hidden Markov Model processing with Expectation-Maximization was used for feature extraction on acquired channel blockades. Classification was then done by Support Vector Machine on five DNA molecules: four DNA hairpin molecules with nine base-pair stem lengths that only differed in their blunt-ended DNA termini, and an eight base-pair DNA hairpin. The accuracy shown is obtained upon completing the 15^th ^single molecule sampling/classification (in approx. 6 seconds), where SVM-based rejection on noisy signals was employed.

All data analyzed is obtained from a nanopore detector and relates to single molecule blockades of a single protein channel. The protein channel is the α-hemolysin pore-forming toxin from *Staphylococcus aureus*, which has a molecule-sized channel opening for partial capture, if not translocation, of biomolecules drawn in by electrophoretic forces (such as DNA) [3,10-20]. Further details on the detector and signal processing architecture are shown in Fig. 2. Further detail on the components of the extracted SVM feature vectors (on events due to individual blockade events), are given in the Methods. Although the figure can only show one SVM classifier implementation (that used in [3]), the data sets examined by all the SVMs described are kept the same (for comparative purposes), so the signal acquisition and feature extraction stages show how the SVM feature vectors are obtained.

### Information measures

The fundamental information measures are Shannon entropy, mutual information, and relative entropy (also known as the Kullback-Leibler divergence or distance). Shannon entropy, **σ = -Σ**_**x**_**p(x)log(p(x))**, is a measure of the information in distribution **p(x)**. Mutual Information, **μ = Σ**_**x**_**Σ**_**y**_**p(xy)log(p(xy)/p(x)p(y))**, is a measure of information one random variable has about another random variable. Relative Entropy (Kullback-Leibler distance): **ρ = Σ**_**x **_**p(x) log(p(x)/q(x))**, is a measure of distance between two probability distributions. Mutual information is a special case of relative entropy between a joint probability (two-component in simplest form) and the product of component probabilities.

### Khinchin derivation of Shannon entropy

In his now famous 1948 paper, Claude Shannon [21] provided a qualitative measure for entropy in connection with communication theory. The Shannon entropy measure was later put on a more formal footing by A. I. Khinchin in an article where he proves that with certain reasonable assumptions the Shannon entropy is unique [22]. A statement of the theorem is as follows:

#### Khinchine Uniqueness Theorem

Let **H(p**_**1**_**,p**_**2**_**,...,p**_**n**_**) **be a function defined for any integer **n **and for all values **p**_**1**_,**p**_**2**_**,...,p**_**n **_such that **p**_**k**_**≥0 (k = 1,2,...,n)**, and **Σ**_**k**_**p**_**k**_** = 1**. If for any function **n **this function is continuous with respect to its arguments, and if the function obeys the three properties listed below, then **H(p**_**1**_**,p**_**2**_**,...,p**_**n**_**) = -λΣ**_**k**_**p**_**k**_**log(p**_**k**_**)**, where **λ **is a positive constant (with Shannon entropy recovered for convention **λ = 1**). The three properties are:

(1) For given **n **and for **Σ**_**k**_**p**_**k**_** = 1**, the function takes its largest value for **p**_**k**_** = 1/n (k = 1,2,...,n**). This is equivalent to Laplace's principle of insufficient reason, which says if you don't know anything assume the uniform distribution (also agrees with Occam's Razor assumption of minimum structure).

(2) **H(ab) = H(a) + H**_**a**_**(b)**, where **H**_**a**_**(b) = -Σ**_**a**_**p(a)log(p(b|a))**, is the conditional entropy. This is consistent with **H(ab)=H(a)+H(b)**, for probabilities of **a **and **b **independent, with modifications involving conditional probability being used when not independent.

(3) **H(p**_**1**_**,p**_**2**_**,...,p**_**n**_**,0) = H(p**_**1**_**,p**_**2**_**,...,p**_**n**_**)**. This reductive relationship, or something like it, is implicitly assumed when describing any system in "isolation."

#### Relative Entropy Uniqueness

This falls out of a geometric formalism on families of distributions: the Information Geometry formalism described by S. Amari [23-25]. Together with Laplace's principle of insufficient reason on the choice of "reference" distribution in the relative entropy expression, this will reduce to Shannon entropy, and thus uniqueness on Shannon entropy from a geometric context. The parallel with geometry is the Euclidean distance for "flat" geometry (simplest assumption of structure), vs. the "distance" between distributions as described by the Kullback-Leibler divergence.

### The Success of Distributions of Nature suggests Generalization from Geometric Feature-Space Kernels to Distribution Feature-Space Kernels

Using the Shannon entropy measure it is possible to derive the classic probability distributions of statistical physics by maximizing the Shannon measure subject to appropriate linear momentum constraints. Constrained variational optimizations involving the Shannon entropy measure can, thus, provide a unified framework with which to describe all, or most, of statistical mechanics. The distributions derivable within the maximum entropy formalism include the Maxwell-Boltzmann, Bose-Einstein, Fermi-Dirac, and Intermediate distributions. The maximum entropy method for defining statistical mechanical systems has been extensively studied by [26].

Both statistical estimation and maximum entropy estimation are concerned with drawing inferences from partial information. The maximum entropy approach estimates a probability density function when only a few moments are known (where there are an infinite number of higher moments). The statistical approach estimates the density function when only one random sample is available out of an infinity of possible samples. The maximum entropy estimation may be significantly more robust (against over-fitting, for example) in that it has an Occam's Razor argument that "cuts both ways" – use *all *of the information given and avoid using any information not given. This means that out of all of the probability distributions consistent with the set of constraints, choose the one that has maximum uncertainty, i.e., maximum entropy [27].

At the same time that Jaynes was doing his work, essentially an optimization principle based on Shannon entropy, Soloman Kullback was exploring optimizations involving a notion of probabilistic distance known as the Kullback-Leibler distance, referred to above as the relative entropy [28]. The resulting minimum relative entropy (MRE) formalism reduces to the maximum entropy formalism of Jaynes when the reference distribution is uniform. The information distance that Kullback and Leibler defined was an oriented measure of "distance" between two probability distributions. The MRE formalism can be understood to be an extension of Laplace's *Principle of Insufficient Reason *(e.g., if nothing known assume the uniform distribution) in a manner like that employed by Khinchine in his uniqueness proof, but now incorporating constraints.

In their book *Entropy Optimization Principles with Applications *[27], Kapur and Kesavan argue for a generalized entropy optimization approach to the description of distributions. They believe every probability distribution, theoretical or observed, is an entropy optimization distribution, i.e., it can be obtained by maximizing an appropriate entropy measure, or by minimizing a relative entropy measure with respect to an appropriate *a priori *distribution. The primary objective in such a modeling procedure is to represent the problem as a simple combination of probabilistic entities that have a simple set of moment constraints. Generalized measures of distributional distance can also be explored along the lines of generalized measures of geometric distance. In physics, not every geometric distance is of interest, however, since the special theory of relativity tells us that spacetime is locally flat (Lorentzian, which is Euclidean on spatial slices), with metric generalization the Riemannian metrics. Likewise, perhaps not all distributional distance measures are created equal either. What the formalism of Information Geometry [23-25] reveals, among other things, is that relative entropy is uniquely structureless (like flat geometry) and is perturbatively stable, i.e., has a well-defined Taylor expansion at short divergence range, just like the locally Euclidean metrics at short distance range.

## Results

### SVM Kernel/Algorithm Variants

**Figure 3:**
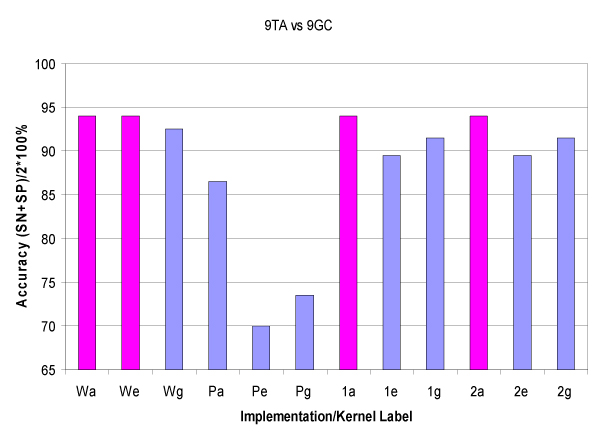
Comparative results are shown on performance of Kernels and algorithmic variants. The classification is between two DNA hairpins (in terms of features from the blockade signals they produce when occluding ion flow through a nanometer-scale channel). Implementations: WH SMO (W); Platt SMO (P); Keerthi1 (1); and Keerthi2 (2). Kernels: Absdiff (a); Entropic (e); and Gaussian (g). The best algorithm/kernel on this and other channel blockade data studied has consistently been the WH SMO variant and the Absdiff and Entropic Kernels. Another benefit of the WH SMO variant is its significant speedup over the other methods (about half the time of Platt SMO and one fourth the time of Keerthi 1 or 2).

The SVM Kernels of interest are "regularized" distances or divergences, where they are regularized if in the form of an exponential with argument the negative of some distance-measure squared (d^2^(x,y)) or symmetrized divergence measure (D(x,y)), the former if using a geometric heuristic for comparison of feature vectors, the latter if using a distributional heuristic. For the Gaussian Kernel: d^2^(x,y) = Σk(xk-yk)^2^; for the Absdiff Kernel d^2^(x,y)=(Σk|xk-yk|)^1/2^; and for the Symmetrized Relative Entropy Kernel D(x,y)= D(x||y)+D(y||x), where D(x||y) is the standard relative entropy. Results are shown in Fig. 3.

The SVM algorithm variants being explored are only briefly mentioned here. In the standard Platt SMO algorithm, η = 2*K12-K11-K22, and speedup variations are described to avoid calculation of this value entirely. A middle ground is sought with the following definition "η = 2*K12-2; If (η >= 0) { η = -1;}" (labeled WH SMO in Fig. 3, underflow handling and other implementations differ slightly in the implementation shown as well).

### SVM-Internal Speedup via differentiating BSVs and SVs

**Figure 4:**
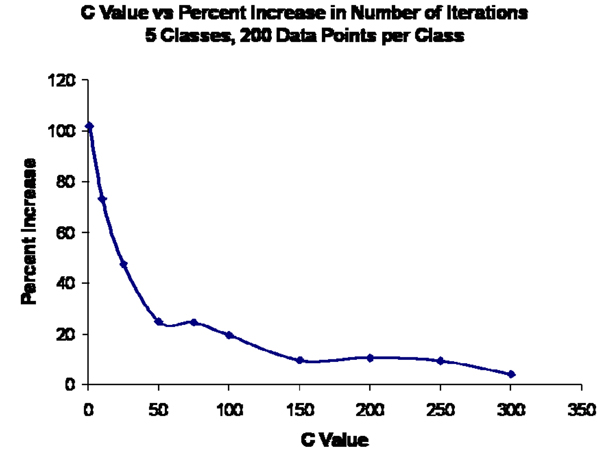
The percent increase in iterations-to-convergence against the 'C' value. For very low values of 'C' the gain is doubled while for very large values of 'C' the gain is low (almost constant for C > 150). Thus we note the dependence of the gain on 'C' value.

**Figure 5:**
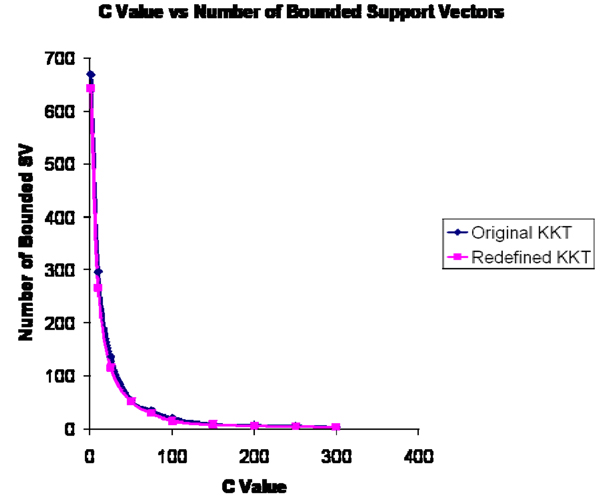
The number of bounded support vectors (BSV) as a function of 'C' value. There are many BSVs for very low values of 'C' and very few BSVs for large values of 'C'. Thus we can say that the number of BSVs plays a vital role in the speed of convergence of the algorithm.

Fig. 4 shows the percent increase in iterations-to-convergence against the 'C' value. Fig. 5 shows the number of bounded support vectors (BSV) as a function of 'C' value. Since the algorithm presented in [7] does not differentiate between SV and BSV, a lot of time is spent in trying to adjust the weights of the BSV i.e. weak data. The weight of a BSV may range from [0, 0.5) in their algorithm. In our modification to the algorithm, shown below, as soon as we identify the BSV (as specified by Case III conditions), its weight is no longer adjusted. Hence faster convergence is achieved without sacrificing accuracy:

For the BSV/SV-tracking speedup, the KKT violators are redefined as:

For all m ≠ y_i _we have:

α_i_^m^{f_yi _- f_m _- 1 + ζ_i_} ≥ 0

Subject to: 1 ≥ α_i_^m ^≥ 0; ∑_m_α_i_^m ^= 1;ζ_i _≥ 0 for all i,m

Where f_m _= (1/β)[w_m_.x_i _+ b_m_] for all m

Case I:

If α_i_^m ^= 0 for m S.T f_m _= f_m_^max^

Implies α_i_^yi ^> 0 and hence ζ_i _= 0

Hence f_yi _- f_m_^max ^- 1 ≥ 0

Case II:

If 1 > α_i_^m ^> 0 for m S.T f_m _= f_m_^max ^and α_i_^yi ^> α_i_^m^

Implies ζ_i _= 0

Hence f_yi _- f_m_^max ^- 1 = 0

Case III:

If 1 ≥ α_i_^m ^> 0 for m S.T f_m _= f_m_^max ^and α_i_^yi ^≤ α_i_^m^

Implies ζ_i _> 0

Hence f_yi _- f_m_^max ^- 1 + ζ_i _= 0

Or f_yi _- f_m_^max ^- 1 < 0

### Data Rejection Tuning with SVM-Internal vs SVM-External Classifiers

**Figure 6:**
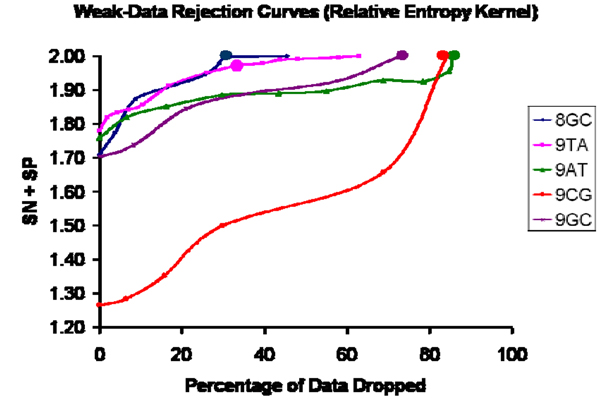
The Percentage Data Rejection vs SN+SP curves are shown for test data classification runs with a binary classifier with one molecule (the positive, given by label) versus the rest (the negative). Since the signal calling wasn't passed through a Decision Tree, it doesn't accurately reflect total throughput, and they don't benefit from the "shielding" shown in the Decision Tree in Fig. 1 prototype. The Relative Entropy Kernel is shown because it provided the best results (over Gaussian and Absdiff).

The SVM Decision Tree shown in Fig. 2b obtained nearly perfect sensitivity and specificity, with a high data rejection rate, and a highly non-uniform class signal-calling throughput. In Fig. 6, the Percentage Data Rejection vs SN+SP curves are shown for test data classification runs with a binary classifier with one molecule (the positive, given by label) versus the rest (the negative). Since the signal calling wasn't passed through a Decision Tree, the way these curves were generated, they don't accurately reflect total throughput, and they don't benefit from the "shielding" shown in the Decision Tree in Fig. 2b prototype. In the SVM Decision Tree implementation described in Fig. 2b[3], this is managed more comprehensively, to arrive at a five-way signal-calling throughput at the furthest node of 16% (in Fig. 1a, 9CG and 9AT have to pass to the furthest node to be classified), while the best throughput, for signal calling on the 8GC molecules, is 75%.

**Figure 7:**
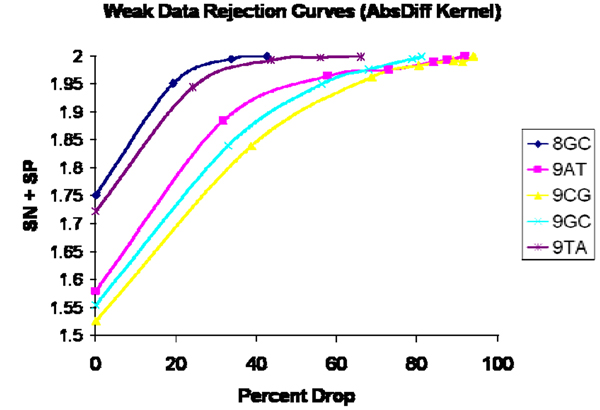
The Percentage Data Rejection vs SN+SP curves are shown for test data classification runs with a multiclass discriminator. The following criterion is used for dropping weak data: for any data point x_i_; if max_m_{f_m_(x_i_)} ≤ Confidence Parameter, then the data point x_i _is dropped. For this data set using AbsDiff kernel (σ^2 ^= 0.2) performed best, and a confidence parameter of 0.8 achieve 100% accuracy.

The SVM Decision Tree classifier's high, non-uniform, rejection can be managed by generalizing to a collection of Decision Trees (with different species at the furthest node). The problem is that tuning and optimizing a single decision tree is already a large task, even for five species (as in Fig. 2). With a collection of trees this problem is seemingly compounded, but can actually be lessened in some ways in that now each individual tree need not be so well-tuned/optimized. Although more complicated to implement than an SVM-External method, the SVM-Internal multiclass methods are not similarly fraught with tuning/optimization complications. Fig. 7 shows the Percentage Data Rejection vs SN+SP curves on the same train/test data splits as used for Fig. 6, except now the drop curves are to be understood as *simultaneous *curves (not sequential application of such curves as in Fig. 6). Thus, comparable, or better, performance is obtained with the multiclass-internal approach and with far less effort since there is no managing and tuning of Decision Trees. Another surprise, and even stronger argument for the SVM-Internal approach to the problem, is that a natural drop zone is indicated by the margin.

### Marginal Drop with SVM-Internal

Suppose we define the criteria for dropping weak data as the margin: For any data point x_i_; let max_m_{f_m_(x_i_)} = f_yi_, and Let f_m _= max_m_{f_m_(x_i_)} for all m ≠ yi, then we define the margin as: (f_yi _- f_m_), hence data point x_i _is dropped if (f_yi _- f_m_) = Confidence Parameter. (For this data set using Gaussian, AbsDiff & Sentropic kernel, a confidence parameter of at least (0.00001)*C was required to achieve 100% accuracy.) The results are shown in Table 1. Using the margin drop approach, there is even less tuning, and there is improved throughput (approximately 75% for *all *species).

### SVM-Internal Clustering

The SVM-Internal approach to clustering was originally defined by [29]. Data points are mapped by means of a kernel to a high dimensional feature space where we search for the minimal enclosing sphere. In what follows, Keerthi's method is used to solve the dual (see Methods for further details).

The minimal enclosing sphere, when mapped back into the data space, can separate into several components; each enclosing a separate cluster of points. The width of the kernel (say Gaussian) controls the scale at which the data is probed while the soft margin constant helps to handle outliers and over-lapping clusters. The structure of a dataset is explored by varying these two parameters, maintaining a minimal number of support vectors to assure smooth cluster boundaries.

We have used the algorithm defined in [29] to identify the clusters, with methods adapted from [30,31 for their handling. If the number of data points is 'n', then we require n(n-1)/2 number of comparisons. We have made modifications to the algorithm such that we eliminate comparisons that do not have an impact on the cluster connectivity. Hence the number of comparisons required will be less than n(n-1)/2.

In each comparison we sub-divide the line segment connecting the two data points into 20 parts; hence we obtain 19 different points on this line segment. The two data points belong to the same cluster only if all the 19 points lie inside the cluster. Given the cost of evaluating utmost 19 points for every comparison, the need to eliminate comparisons that do not have an impact on the cluster connectivity becomes even more important. Finally we have used Depth First Search (DFS) algorithm for the cluster harvest. Results are shown in Tables 2 and 3. The approach to the solving the Dual problem is shown in the Methods.

### SVM-External Clustering

**Figure 8:**
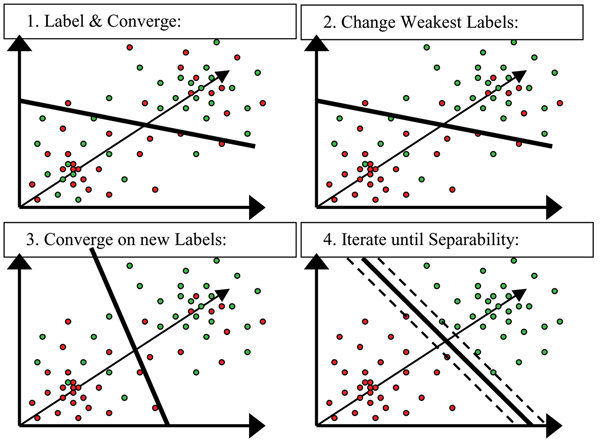
Shown is the schematic for an "external" SVM clustering algorithm.

**Figure 9:**
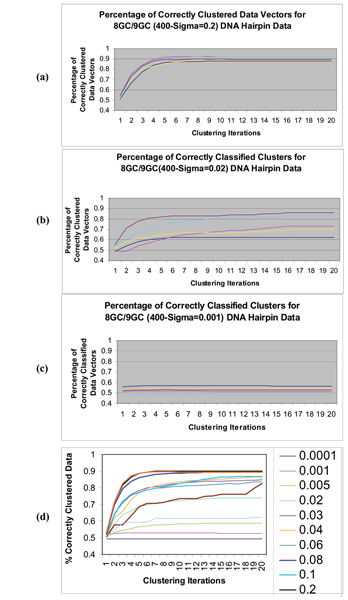
**(a) **The percentage correct classification (an indication of the clustering success) is shown with successive iteration of the clustering algorithm. Five separate test runs are shown, on different data from the same classes. Note that the plateau at around 0.9, this is approximately the performance of a supervised binary SVM on the same data (i.e., perfect separation isn't possible with this data without employing weak-data rejection). **(b) **The degradation in clustering performance for less optimal selection of kernel and tuning parameter (variance in case of Gaussian). **(c) **The degradation in clustering performance for non-optimal selection of kernel and tuning parameter (variance in case of Gaussian). **(d) **Summary of the degradation in clustering performance for less optimal selection of kernel and tuning parameter – with averages of the five test-runs are used as representative curves for that kernel/tuning selection in the above.

**Figure 10:**
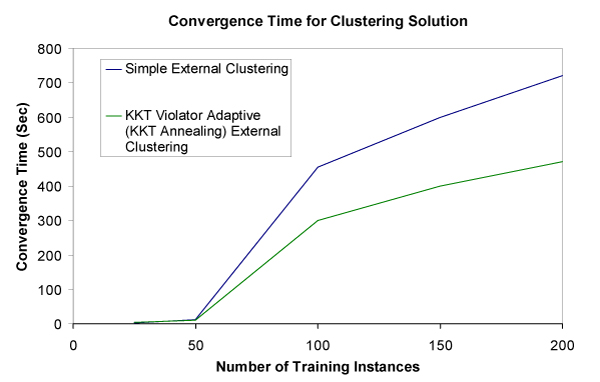
Efforts are underway to use simulated annealing in the number of KKT Violators tolerated on each iteration of the external clustering algorithm, to accelerate the convergence (clustering) process. Our current approach, results shown, approximately halves the cluster time needed.

As with the multiclass SVM discriminator implementations, the strong performance of the binary SVM enables SVM-External as well as SVM-Internal approaches to clustering. Our external-SVM clustering algorithm clusters data vectors with no *a priori *knowledge of each vector's class. The algorithm works by first running a Binary SVM against a data set, with each vector in the set randomly labeled, until the SVM converges (Fig. 8). In order to obtain convergence, an acceptable number of KKT violators must be found. This is done through running the SVM on the randomly labeled data with different numbers of allowed violators until the number of violators allowed is near the lower bound of violators needed for the SVM to converge on the particular data set. Choice of an appropriate kernel and an acceptable sigma value also will affect convergence. After the initial convergence is achieved, the sensitivity + specificity will be low, likely near 1. The algorithm now improves this result by iteratively relabeling the worst misclassified vectors, which have confidence factor values beyond some threshold, followed by rerunning the SVM on the newly relabeled data set. This continues until no more progress can be made. Progress is determined by an increasing value of sensitivity + specificity, hopefully nearly reaching 2. After this process, a high percentage of the previously unknown class labels of the data set will be known. With sub-cluster identification upon iterating the overall algorithm on the positive and negative clusters identified (until the clusters are no longer separable into sub-clusters), this method provides a way to cluster data sets without prior knowledge of the data's clustering characteristics, or the number of clusters. Figures 9 and 10 show clustering runs on a data set with a mixture of 8GC and 9GC DNA hairpin data. The set consists of 400 elements. Half of the elements belong to each class. The SVM uses a Gaussian Kernel and allows 3% KKT Violators.

### Machine Learning and Cheminformatics Tools are Accessible via Website

**Figure 11:**
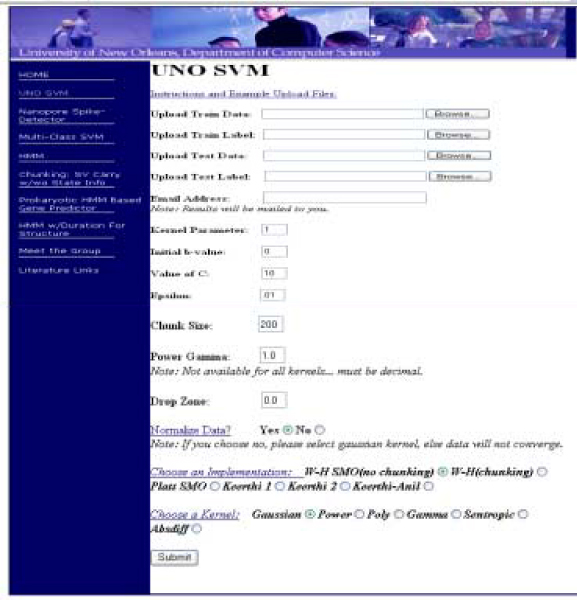
Several channel current cheminformatics tools are available for use via web interfaces at . These tools include a variety of SVM interfaces for classification and clustering (binary and multiclass), and HMM tools for feature extraction and structure identification (with applications to both channel current cheminformatics and computational genomics).

The web-site provides an interface to several binary SVM variants (with other novel kernel selections), to a multiclass (internal) SVM, an FSA-based nanopore spike detector, and an HMM-based channel current feature extraction. New, web-accessible, channel current analysis tools, have also been developed for kinetic feature extraction (via channel current sub-level lifetimes), and clustering. The website is designed using HTML and CGI scripts that are executed to process the data sent when a form filled in by the user is received at the web server – results are then e-mailed to the address indicated by the user. The interface to this and all other software described is available via the group Home Page:  (see Fig. 11). The SVM interface offers options on chunk processing for large training sets (SV-carry by appending to next training chunk and SV-carry by maintaining state and injecting ("unfreezing") the next training chunk (a specialized α-heuristic). The interface offers use of arbitrary or structured feature vectors – where structured, in this case, corresponds to feature vector components that satisfy the properties of a non-trivial, non-reducible, discrete probability distribution. There is an SVM interface for a new single-optimization multiclass SVM discriminator (it simultaneously optimizes multiple hyperplanes). There is also an interface for our SVM-based clustering methods.

## Discussion

### Adaptive Feature Extraction/Discrimination

Adaptive feature extraction and discrimination, in the context of SVMs, can be accomplished by small batch reprocessing using the learned support vectors together with the new information to be learned. The benefit is that the easily deployed properties of SVMs can be retained while at the same time co-opting some of the on-line adaptive characteristics familiar from on-line learning with neural nets. This is also compatible with the chunking processing that is already implemented. A situation where such adaptation might prove necessary in nanopore signal analysis is if the instrumentation was found to have measurable, but steady, drift (at a new level of sensitivity for example). At the forefront of online adaptation, where the discrimination and feature extraction optimizations are inextricably mixed, further progress may derive benefit from the Information-Geometrical methods of S. Amari [23-25].

### Robust SVM performance in the presence of noise

In a parallel datarun to that indicated in Fig. 2a, with 150 component feature vectors, feature vectors with the full set of 2600 components were extracted (i.e., no compression was employed on the transition probabilities). SVM performance on the same train/test data splits, but with 2600 component feature vectors instead of 150 component feature vectors, offered similar performance after drop optimization. This demonstrates a significant robustness to what the SVM can "learn" in the presence of noise (some of the 2600 component have richer information, but even more are noise contributors).

### AdaBoost Feature Selection

If SVM performance on the full HMM parameter set (the features extracted for each blockade signal) offers equivalent performance after rejecting weak data, then the possibility for significant improvement with selection on good parameters. An AdaBoost method is being used to select HMM parameters by representing each feature vector component as an independent Naïve Bayes classifier (trained on the data given), that then comprise the pool of experts in the AdaBoost algorithm [32-34]. The experts AdaBoost assigns heaviest weighting will then the components selected in the new, AdaBoost assigned, feature vector compression.

## Conclusion

• External Multi-class SVM gave best results with Sentropic Kernel while Internal Multi-class SVM gave best results with AbsDiff kernel.

• Internal Multi-class approach overcomes the need to search for the best performing tree out of many possibilities. This is a huge advantage especially when the number of classes is large.

• Using a margin to define the drop zone for the internal multi-class approach produced far better results i.e. fewer data were dropped to achieve 100% accuracy.

• Additional benefit of using the margin is that the drop zone tuning to achieve 100% accuracy becomes trivial.

• External and Internal SVM *Clustering *Methods were also examined. The results show that our SVM-based clustering implementations can separate data into proper clusters without any prior knowledge of the elements' classification. this can be a powerful resource for insight into data linkages (topology).

## Methods

### The Feature Extraction used to obtain the Feature Vectors for SVM analysis

#### Signal Preprocessing Details

The Nanopore Detector is operated such that a stream of 100 ms samplings are obtained (throughput was approximately one sampling per 300 ms in [3]). Each 100 ms signal acquired by the time-domain FSA consists of a sequence of 5000 sub-blockade levels (with the 20 μs analog-to-digital sampling). Signal preprocessing is then used for adaptive low-pass filtering. For the data sets examined, the preprocessing is expected to permit compression on the sample sequence from 5000 to 625 samples (later HMM processing then only required construction of a dynamic programming table with 625 columns). The signal preprocessing makes use of an off-line wavelet stationarity analysis (Off-line Wavelet Stationarity Analysis, Figure 2b, also see [35]).

#### HMMs and Supervised Feature Extraction Details

With completion of preprocessing, an HMM [36] is used to remove noise from the acquired signals, and to extract features from them (Feature Extraction Stage, Fig. 2b). The HMM is, initially, implemented with fifty states, corresponding to current blockades in 1% increments ranging from 20% residual current to 69% residual current. The HMM states, numbered 0 to 49, corresponded to the 50 different current blockade levels in the sequences that are processed. The state emission parameters of the HMM are initially set so that the state j, 0 <= j <= 49 corresponding to level L = j+20, can emit all possible levels, with the probability distribution over emitted levels set to a discretized Gaussian with mean L and unit variance. All transitions between states are possible, and initially are equally likely. Each blockade signature is de-noised by 5 rounds of Expectation-Maximization (EM) training on the parameters of the HMM. After the EM iterations, 150 parameters are extracted from the HMM. The 150 feature vector components are extracted from the 50 parameterized emission probabilities, a 50-element compressed representation of the 50^2 ^transition probabilities, and an *a posteriori *information from the Viterbi path solution which is, essentially, a de-noised histogram of the bloackade sub-level occupation probabilities (further details in [3]). This information elucidates the blockade levels (states) characteristic of a given molecule, and the occupation probabilities for those levels, but doesn't directly provide kinetic information. An HMM-with-Duration has recently been introduced to better capture the latter information, but such feature vectors are not used in the studies shown in this paper, so this approach isn't discussed further in this paper.

### Solving the Dual (Based on Keerthi's SMO [37])

The dual formalism is: 1 - ∑_i,j_β_i_β_j_K_ij _where 0 ≤ β_i _≤ C; K_ij _= exp(-||x_i _- x_j_||^2^/2σ^2^), also ∑_i_β_i _= 1. For any data point 'x_k_', the distance of its image in kernel space from the center of the sphere is given by: R^2^(x_k_) = 1 - 2∑_i_β_i_K_ik _+ ∑_i,j_β_i_β_j_K_ij_. The radius of the sphere is R = {R(x_k_) | x_k _is a Support Vectors}, hence data points which are Support Vectors lie on cluster boundaries. Outliers are points that lie outside of the sphere and therefore they do not belong to any cluster i.e. they are Bounded Support Vectors. All other points are enclosed by the sphere and therefore they lie inside their respective cluster. KKT Violators are given as: (i) If 0 < β_i _< C and R(x_i_) ≠ R; (ii) If β_i _= 0 and R(x_i_) > R; and (iii) If β_i _= C and R(x_i_) < R.

The Wolfe dual is: f(β) = Min _β _{∑_i,j_β_i_β_j_K_ij _- 1}. In the SMO decomposition, in each iteration we select β_i _& β_j _and change them such that f(β) reduces. All other β's are kept constant for that iteration. Let us denote β_1 _& β_2 _as being modified in the current iteration. Also β_1 _+ β_2 _= (1 - ∑_i = 3_β_i_) = s, a constant. Let ∑_i = 3_β_i_K_ik _= C_k_, then we obtain the SMO form: f(β_1_,β_2_) = β^2^_1 _+ β^2^_2 _+ ∑_i,j = 3_β_i_β_j_K_ij _+ 2β_1_β_2_K_12 _+ 2β_1_C_1 _+ 2β_2_C_2_. Eliminating β_1_: f(β_2_) = (s - β_2_)^2 ^+ β^2^_2 _+ ∑_i,j = 3_β_i_β_j_K_ij _+ 2(s - β_2_)β_2_K_12 _+ 2(s - β_2_)C_1 _+ 2β_2_C_2_. To minimize f(β_2_), we take the first derivative w.r.t. β_2 _and equate it to zero, thus f'(β_2_) = 0 = 2β_2_(1 - K_12_) - s(1 - K_12_) - (C_1 _- C_2_), and we get the update rule: β_2_^new ^= [(C_1 _- C_2_)/2(1 - K_12_)] + s/2. We also have an expression for "C_1 _- C_2_" from: R(x_1_^2^) - R(x_2_^2^) = 2(β_2 _- β_1_)(1 - K_12_) - 2(C_1 _- C_2_), thus C_1 _- C_2 _= [R(x_2_^2^) - R(x_1_^2^)]/2 + (β_2 _- β_1_)(1 - K_12_), substituting, we have:

β_1_^new ^= β_1_^old ^- [R(x_2_^2^) - R(x_1_^2^)]/[4(1 - K_12_)]

#### Keerthi Algorithm

Compute 'C': if percent outliers = n and number data points = N, then: C = 100/(N*n)

Initialize β: Initialize m = int(1/C) - 1 number of randomly chosen indices to 'C'

Initialize two different randomly chosen indices to values less than 'C' such that ∑_i_β_i _= 1

Compute R^2^(x_i_) for all 'i' based on the current value of β.

Divide data into three sets: Set I if 0 < β_i _< C; Set II if β_i _= 0; and Set III if β_i _= C.

Compute R^2^_low = Max{ R^2^(x_i_) | 0 ≤ β_i _< C} and R^2^_up = Min{ R^2^(x_i_) | 0 < β_i _≤ C}.

In every iteration execute the following two paths alternatively until there are no KKT violators:

1. Loop through all examples (call Examine Example subroutine)

Keep count of number of KKT Violators.

2. Loop through examples belonging only to Set I (call Examine Example subroutine) until R^2^_low - R^2^_up < 2*tol.

Examine Example Subroutine

a. Check for KKT Violation. An example is a KKT violator if:

Set II and R^2^(x_i_) > R^2^_up; choose R^2^_up for joint optimization

Set III and R^2^(x_i_) < R^2^_low; choose R^2^_low for joint optimization

Set I and R^2^(x_i_) > R^2^_up + 2*tol OR R^2^(x_i_) < R^2^_low - 2*tol; choose R^2^_low or R^2^_up for joint optimization depending on which gives a worse KKT violator

b. Call the Joint Optimization subroutine

Joint Optimization Subroutine

a. Compute η = 4(1 - K_12_) where K_12 _is the kernel evaluation of the pair chosen in Examine Example

b. Compute D = [R^2^(x_2_) - R^2^(x_1_)]/η

c. Compute Min{(C - β_2_), β_1_} = L1

d. Compute Min{(C - β_1_), β_2_} = L2

e. If D > 0; then D = Min{D, L1}

Else D = Max{D, -L2}

f. Update β_2 _as: β_2 _= β_2 _+ D

g. Update β_1 _as: β_1 _= β_1 _- D

h. Re-compute R^2^(x_i_) for all 'i' based on the changes in β_1 _& β_2_

i. Re-compute R^2^_low & R^2^_up based on elements in Set I, R^2^(x_1_) & R^2^(x_2_)

### The SVM-External Clustering Method

The SVM-clustering software is written in Perl. It runs data on a separate Binary SVM also written in Perl. This SVM uses a C file for kernel calculations. The data run on the SVM is created by running raw data through a tFSA/HMM(written in C), which creates a data set that contains 151 feature vectors for each element. The following is a simple step-by-step description of the basic algorithm used for SVM-clustering on this data:

1. Start with a set of data vectors (obtained through running raw data through tFSA/HMM feature extraction in Fig. 2b).

2. Randomly label each vector in the set as positive or negative.

3. Run the SVM on the randomly labeled data set until convergence is obtained (random relabeling is needed if prior random label scheme does not allow for convergence).

4. After initial convergence is obtained for the randomly labeled data set, relabel the misclassified data vectors, which have confidence factor values greater than some threshold.

5. Rerun the SVM on the newly relabeled data set.

6. Continue relabeling and rerunning SVM until no vectors in the data set are misclassified (or there is no improvement).

## Authors' contributions

The paper was written by SWH and AY. The external clustering work was contributed by CM. The channel current feature vector extraction used to create the data sets was performed by ML.

## Figures and Tables

**Table 1:** The table shows the results of dropping data that falls in the margin. For any data point x_i_; let max_m_{f_m_(x_i_)} = f_yi_, and Let f_m _= max_m_{f_m_(x_i_)} for all m ≠ yi, then we define the margin as: (f_yi _- f_m_), hence data point x_i _is dropped if (f_yi _- f_m_) ≤ Confidence Parameter. Using the margin drop approach, there is even less tuning, and there is improved throughput (approximately 75% for *all *species).

**Kernel**	**8GC**	**9AT**	**9CG**	**9GC**	**9TA**
**Gaussian**	P: 1268 TP: 1087 SN+SP: 1.76 P: 1087 TP: 1087 SN+SP: 2 **Drop = 9.42**	P: 1178 TP: 934 SN+SP: 1.57 P: 934 TP: 934 SN+SP: 2 **Drop = 22.17**	P: 1166 TP: 904 SN+SP: 1.53 P: 904 TP: 904 SN+SP: 2 **Drop = 24.67**	P: 1172 TP: 897 SN+SP: 1.51 P: 897 TP: 897 SN+SP: 2 **Drop = 25.25**	P: 1216 TP: 1027 SN+SP: 1.70 P: 1027 TP: 1027 SN+SP: 2 **Drop = 14.42**

**AbsDiff**	P: 1407 TP: 1134 SN+SP: 1.75 P: 1134 TP: 1134 SN+SP: 2 **Drop = 5.5**	P: 1151 TP: 928 SN+SP: 1. 58 P: 928 TP: 928 SN+SP: 2 **Drop = 22.67**	P: 1177 TP: 906 SN+SP: 1.53 P: 906 TP: 906 SN+SP: 2 **Drop = 24.5**	P: 1050 TP: 870 SN+SP: 1.55 P: 870 TP: 870 SN+SP: 2 **Drop = 27.5**	P: 1215 TP: 1040 SN+SP: 1.72 P: 1040 TP: 1040 SN+SP: 2 **Drop = 13.33**

**Entropic**	P: 1165 TP: 1038 SN+SP: 1.75 P: 1038 TP: 1038 SN+SP: 2 **Drop = 13.5**	P: 1480 TP: 995 SN+SP: 1.50 P: 991 TP: 991 SN+SP: 2 **Drop = 17.42**	P: 1348 TP: 922 SN+SP: 1.45 P: 920 TP: 920 SN+SP: 2 **Drop = 23.33**	P: 960 TP: 804 SN+SP: 1.50 P: 803 TP: 803 SN+SP: 2 **Drop = 33.08**	P: 1047 TP: 970 SN+SP: 1.73 P: 970 TP: 970 SN+SP: 2 **Drop = 19.17**

**Table 2:** The table shows clustering predictions when working with 400 Samples (200 each of 9GC & 9CG) with a Gaussian Kernel with Width = 50 (σ^2 ^= 0.01).

**C Value**	**Number of SV**	**Percent of Outliers**	**Number of Clusters**	**Number of Comparisons**
0.25	91	0	10	39005
0.025	87	1.25	5	37020
0.0125	44	13.75	4	29202
0.01	29	21.75	2	24145

**Table 3:** The table shows clustering predictions when working with 1200 Samples (600 each of 9GC & 9CG) with a Gaussian Kernel with Width = 50 (σ^2 ^= 0.01).

**C Value**	**Number of SV**	**Percent of Outliers**	**Number of Clusters**	**Number of Comparisons**
0.00833	106	5.8	4	10873
0.00417	37	18.25	2	232021
0.00333	31	23.8	2	203278
0.00278	23	29.08	2	177533
